# Adequacy of setting standards for kick impact in the Taekwondo electronic scoring system: comparison of a reference group model based on empirical data

**DOI:** 10.1186/s13102-021-00340-x

**Published:** 2021-09-15

**Authors:** Chang-Hwan Choi, Hyeri Oh, Minsoo Jeon

**Affiliations:** 1 College of Education, Department of Physical Education, Kangwon National University, Chuncheon, Korea; 2 Sports Medicine, Cha University, Gyeonggi-do, Korea; 3 Department of Sports Science, Korea National Sport University, Seoul, Korea

**Keywords:** Kick impact, Protector, Scoring systems, Reference group model, Sports analytics, Martial arts

## Abstract

**Background:**

In Taekwondo competitions, the rule is that points are scored when the impact of the kick reaches a predetermined threshold of strength. This study aimed to explore the adequacy of the protector and scoring system (PSS) designed to determine taekwondo body scoring based on a reference group model (RGM). Specifically, the kicking impact of the PSS was calculated using data from 188 matches fought during of 2018 Jakarta Asian Games. The RGM was designed based on empirical data by classifying the victory and defeat groups by gender and weight class, and the scoring method was set according to these criteria.

**Result:**

The result of this study are as follows. First, there was no difference in the average impact of kicks of taekwondo players by weight class. Second, result of setting up the kick scoring impact standards of taekwondo PSS by classifying the winning and non-winning groups, the kick scoring impact set by the WT was found to be high in all weight classes except 58 kg. Lastly, result of comparing the settings of impact to score according to weight classes, the kick scoring impact standard set by the WT was higher in heavyweight (men's: under 80 kg, + 80 kg, women's: under 67 kg, over 67 kg) than in the lightweight (men's: under 58 kg, under 63 kg, under 68 kg, women's: under 49 kg, under 53 kg, under 57 kg).

**Conclusion:**

The kick scoring impact set by the WT was found to be high in all weight classes except for the under 58 kg class defining kick scoring impact based on the standards of WT-certified PSS by classifying the matches into winning and non-winning groups. Finally, as a result of comparing the scoring impact settings according to weight class, the kick scoring impact standard set by the WT was higher for the heavier weight classes than for the lighter weight classes.

## Background

Taekwondo is a sport that attracts many people and it is becoming a popular sport practiced worldwide. Furthermore, taekwondo was adopted as an Olympic sport where only the best athletes participate. In this regard, the World Taekwondo (WT) is making various efforts to improve the sport of taekwondo, for example by assessing the fairness of the scoring system which is a currently an issue in taekwondo competitions.

Changes in the scoring method are being implemented to ensure fairness in taekwondo matches. In the past, the scores were determined based on the subjective judgment of the referees [1]. However, the protector and scoring system (PSS) was developed and introduced in order to address problems with the scoring method encountered in the past. PSS sets scoring criteria according to the kick impact determined in advance for male and female weight classes, and automatically calculates the score when the scoring area is hit harder than the set kick impact threshold [14]. The benefit of setting a kick impact scoring threshold is that the higher the weight class, the higher the kick impact standard. In this regard, taekwondo is applying a more objective and consistent scoring tool than in the past [2], contributing to its becoming a global sport.

However, the taekwondo PSS can present several problems despite its advantages. Furthermore, the validity of the kick impact scoring standard remains unclear, even though its objectivity and consistency were improved. Currently, the WT has arbitrarily set the kick impact scoring scale for male and female weight classes under an expert's subjective judgment [9]. Furthermore, it is necessary to establish the basis for different impact settings, according to weight class. For example, in the case of males, the scoring impact of the under 58 kg weight class was set to 18 levels, that of the under 63 kg weight class was set to 20 levels, and that of the under 68 kg weight class was set to 21 levels. Despite the same 5 kg difference, the under-58 kg and under 63 kg weight classes have a difference of two levels, while the under 63 kg and under 68 kg weight classes have a difference of one level [3]. These differences in setting kick impact leads to doubt in both the players and the coaches. In fact, a difference of one level is not great, but due to the nature of taekwondo, where the criteria for judging scores are based on a reference-oriented evaluation, it could lead to skewed results. Moreover, studies on setting kick scoring impact are continuously required because it can be an important factor affecting score, game flow, and player results. Research on taekwondo PSS has been conducted on the score sensor and satisfaction with the PSS [4, 5, 7, 12, 13, 15], and these studies have calculated the difficulty of assessing kick impact by weight class [3]. Thus, studies on setting kick scoring impact are required. This study aimed to confirm the adequacy of setting a kick impact standard for taekwondo PSS. Specifically, we analyzed the difference between the kick impact standard set by the WT and the kick impact standard based on kick impact data from the 2018 Jakarta Asian Games. During this process, weverified whether the kicking standard currently applied was high or low.

## Methods

### Research data

In this study, the adequacy of a kick impact standard by weight class was confirmed using 2018 Jakarta Asian Games taekwondo match data. Specifically, there were a total of 104 matches (under 58 kg weight class: 24 matches, under 63 kg weight class: 25 matches, under 68 kg weight class: 21 matches, under 80 kg weight class: 20 matches, and over 87 kg weight class: 14 matches), with a maximum of 26 countries and a minimum of 21 countries. For women's matches, there were a total of 84 matches (under 49 kg weight class: 16 matches, under 53 kg weight class: 17 matches, under 57 kg weight class: 19 matches, under 67 kg weight class: 15 matches, over 67 kg weight class: 17 matches), with a maximum of 26 countries and a minimum of 15 countries (Table 1).

**Table 1:** Taekwondo Men's and Women's matches by weight class and participating country

Men's			Women's		
Weight class	Number of games (%)	Number of participating countries	Weight class	Number of games (%)	Number of participating countries
Under 58 kg	24 (23.1)	26	Under 49 kg	16 (19.1)	17
Under 63 kg	25 (24.0)	26	Under 53 kg	17 (20.2)	18
Under 68 kg	21 (20.2)	22	Under 57 kg	19 (22.6)	20
Under 80 kg	20 (19.2)	21	Under 67 kg	15 (17.9)	16
Over 80 kg	14 (13.5)	21	Over 67 kg	17 (20.2)	15
Total	104 (100)		Total	84 (100)	

### Data acquisition procedure

In this study, data from KP&P PSS obtained in the 2018 Jakarta Asian Games taekwondo matches were provided by the Asian taekwondo Federation and KP&P. The KP&P PSS is an official accreditation body certified by the WT (October 23, 2012) and it is the official scoring system used in world competitions and Asian Games.

Only the scoring impact generated in the trunk area was entered and classified by weight class in order to use the data for the scoring impact calculated by KP&PPSS as research data. Data was input by three taekwondo match analysis experts to minimize error. Several matches were randomly selected and re-recorded to confirm the match with the previous record, after entering the data. The PSS kicking impact calculated through this process was 2,286 times for men (scoring frequency: 814 times, effective hit frequency: 1430), and 1,389 times for women (scoring frequency: 650 times, effective hit frequency: 688 times), and these values were used as research data.

### Group classification for establishing kick impact reference points for the protector and scoring system

The criterion groups model, one of the empirical methods, was used in this study to confirm the adequacy of the taekwondo PSS kick impact reference point. The reference group model is divided into the group that meets the criteria and the on that does not. The intersection point of the distribution of scores of the two groups was set as the reference point [11]. Therefore, two methods were used in this study to classify the group to determine the appropriateness of the kick impact reference. First, the winning group (which met the criteria) and the non-winning group (which did not meet the criteria), according to weight category, were divided and compared with the reference point shown at the intersection point of the 2-group score distributions with the kick impact reference point currently applied by WT. Then, after classifying the groups according to weight class, the score distribution intersection point was identified. For example, for men's weight classes, the current kick impact reference point is determined by checking and comparing the intersection of the score distributions for the two groups in the following order: under 58 kg, and under 63 kg, under 63 kg and under 68 kg, under 68 kg and under 80 kg, under 80 kg, and over 80 kg weight classes.

### Data processing method

The descriptive statistics for the PSS kick scoring impact were calculated by weight class. Cutoff scores were calculated to establish scoring criteria according to the reference group. The cutoff score was calculated based on the standard normal distribution using the mean and standard deviation of the kicking intensities of the winning and non-winning groups according to gender and weight. Furthermore, the intersection of the kicking intensity distributions of the winning and non-winning groups was selected as the cutoff score [6]. The accuracy of the classification was calculated to verify the validity of the scoring criteria through the cutoff score [10]. R program (ver 3.6.1) and MS-Excel were used for data analysis.

## Result

### Basic information on kick force of top taekwondo athletes

**Fig. 1:**
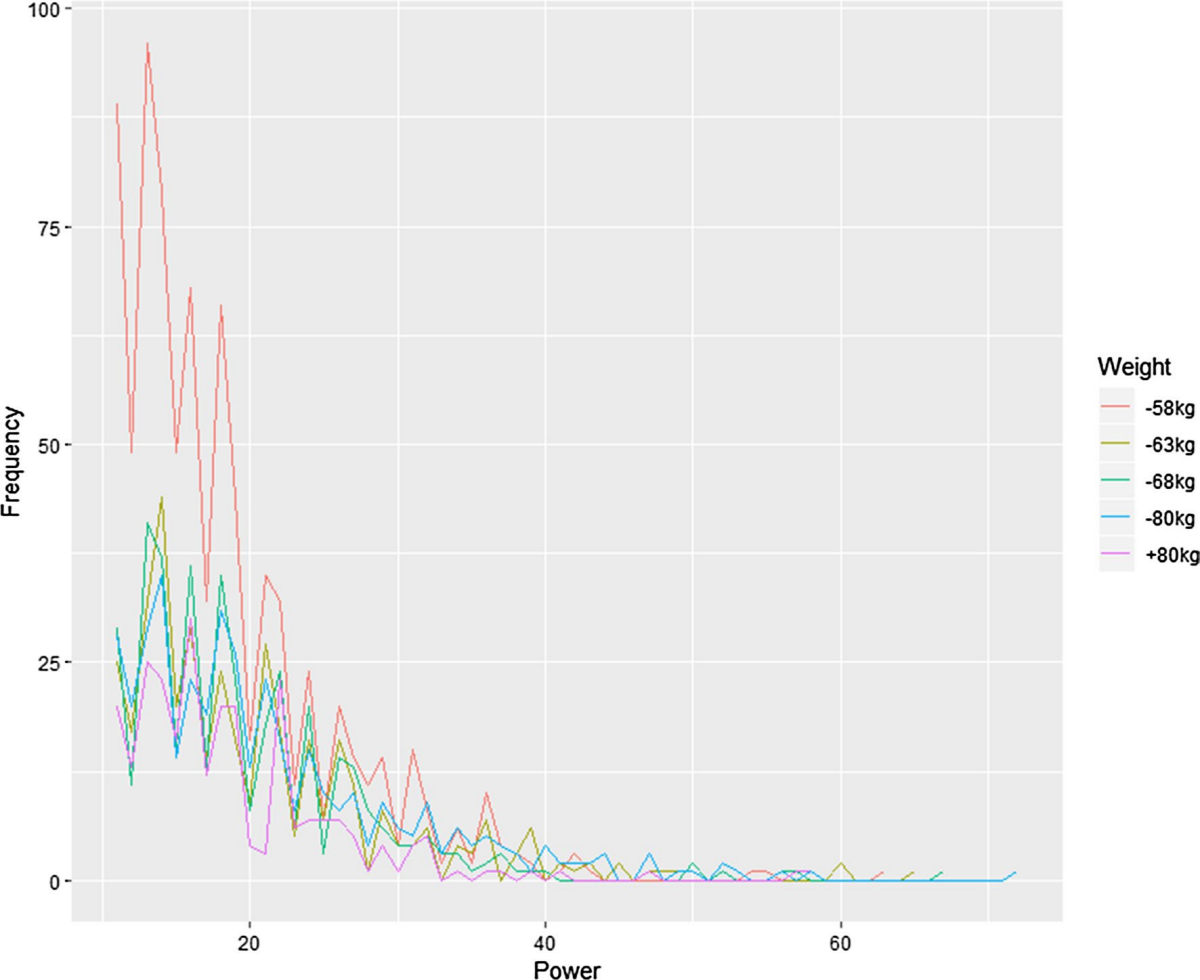
Men's kick impact figures by weight class

**Fig. 2:**
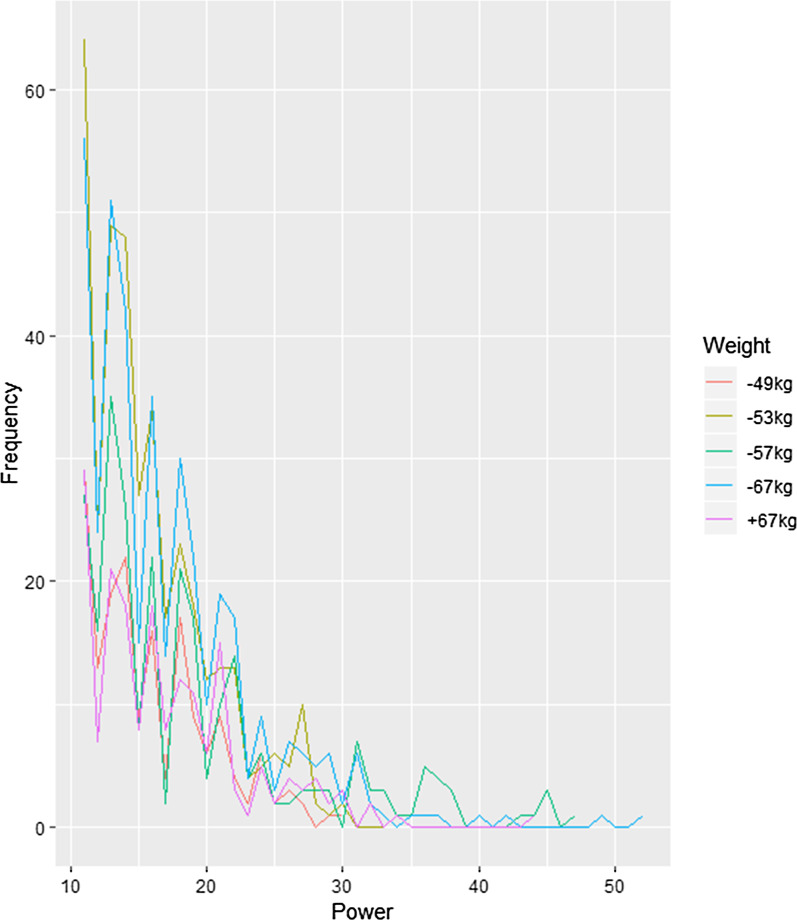
Women's kick impact figures by weight class

Figures 1 and 2 show the graph so the kick scoring impact by gender and weight class. As a result, the incidence of kick impact was high, between 10 to 20 levels, in both the men's and women's divisions. Also, regardless of weight class, the form of kick impact was shown as static distribution. Specifically, as shown in Table 2, for the men's under 58 kg weight class, the average kicking impact was 18.4 levels, and 18.7 levels for the over 80 kg weight class. In the women's division, the average kick impact for the under 49 kg weight class was 16.1 levels, and 17.3 levels for the over 67 kg weight class.

The following graph shows the impact force of kicks in different weight classes. The y-axis represents kick frequency and the x-axis represents impact force.

**Table 2:** Results of protector and scoring system descriptive statistics by gender and weight class (M ± SD)

Men				Women			
Weight class	Scoring group	Effective hit group	Average kicking impact	Weight class	Scoring group	Effective hit group	Average kicking impact
Under 58 kg	24.6 ± 7.17	13.6 ± 1.88	18.4 ± 7.37	Under 49 kg	19.9 ± 3.41	12.7 ± 1.39	16.1 ± 4.41
Under 63 kg	28.3 ± 8.51	14.7 ± 2.40	20.6 ± 8.93	Under 53 kg	21.2 ± 3.95	13.2 ± 1.73	16.0 ± 4.70
Under 68 kg	27.4 ± 7.59	15.1 ± 2.65	19.7 ± 7.82	Under 57 kg	25.4 ± 7.67	13.3 ± 1.71	19.1 ± 8.10
Under 80 kg	32.2 ± 8.62	16.1 ± 3.39	21.4 ± 9.44	Under 67 kg	25.5 ± 5.82	14.3 ± 2.59	17.3 ± 6.17
Over 80 kg	30.9 ± 7.70	16.4 ± 3.68	18.7 ± 6.99	Over 67 kg	27.3 ± 4.37	15.2 ± 3.29	17.3 ± 5.71

Score group: Impact force of kicks that have successfully scored in a match. Effective Hit Group: Impact force of kicks that have landed on the electronic protector without scoring in a match

### Setting and conducting validity tests for the reference points of kick scoring impact for top taekwondo players in the protector and scoring system

#### Setting kick impact reference points by gender and weight classes (classified according to the winning and non-winning groups)

Table 3 shows the criteria for kick impacts in the PSS by gender and weight classes. The groups are divided into winning and non-winning groups in order to set the kick scoring impact criteria. As a result, for males, the under 58 kg weight class was 18 levels (acc: 0.365), the under 63 kg weight class was 19 levels (acc: 0.482), the under 68 kg weight class was 19 levels (acc: 0.494), the under 80 kg weight class was 19 levels (acc: 0.445), and the over 80 kg weight class was 20 levels (acc: 0.496). In the case of the over 80 kg weight class, the classification accuracy was equal to.496 at 19 and 20 levels, but it was determined that it is more reasonable to set the impact to 20 levels when looking at the sensitivity index for validity, where 19 levels: 0.674 and 20 levels: 0.689. In women, the classification accuracy was as follows: under 46 kg at 15 levels (acc: 0.489), under 53 kg at 15 levels (acc: 0.525), under 57 kg at 17 levels (acc: 0.498), under 67 kg at 16 levels (acc: 0.443), and over 67 kg at 18 levels (acc: 0.473). Kick scoring impact, which is divided into the winning and non-winning groups, was set lower than that set by the WT.

**Table 3:** Kick scoring impact setting standards and their validations (group classification based on winning and non-winning groups)

Men					Women				
Weight class	A	B	Validation index		Weight class	A	B	Validation index	
Under 58 kg	18	18.8	18	0.365	Under 49 kg	16	16.0	15	0.489
			19	0.329				16	0.466
			20	0.309				17	0.454
Under 63 kg	20	20.4	19	0.482	Under 53 kg	17	16.0	15	0.525
			20	0.464				16	0.499
			21	0.426				17	0.485
Under 68 kg	21	19.3	18	0.486	Under 57 kg	18	18.0	17	0.498
			19	0.494				18	0.486
			20	0.478				19	0.475
Under 80 kg	23	19.5	19	0.445	Under 67 kg	20	17.3	16	0.443
			20	0.423				17	0.433
			21	0.401				18	0.382
Over 80 kg	25	19.0	18	0.458	Over 67 kg	22	16.8	17	0.462
			19	0.496				18	0.473
			20	0.496				19	0.467

A: Kick impact standards officially established by the World Taekwondo Federation. B: Kick impact standards set in this study

#### Setting men's and women's kick impact reference points (by weight classes)

Table 4 shows the kick impact criteria of the PSS according to men's and women's weight classes. Weight class was divided based on weight class in order to set the kick scoring impact. As a result, for men under 58 kg and under 63 kg, 21 levels (acc: 0.622), under 63 kg and under 68 kg, 21 levels (acc: 0.492), under 68 kg and under 80 kg, 22 levels, and under 80 kg and over 80 kg, 21 levels (acc: 0.502). For women, under 49 kg and under 53 kg, 15 levels (acc: 0.468), under 53 kg and under 57 kg, 19 levels (acc: 0.610), under 57 kg and under 67 kg, 17 levels (acc: 0.448), and under 67 kg and over 67 kg, 18 levels (acc: 0.572). When dividing the kick scoring impact into weight classes, it was found to be higher than the kick scoring impact currently applied in most heavyweight classes.

**Table 4:** Kick scoring impact setting standards and their validations (group classification based on weight class)

Men					Women				
Weight class	A	B	Validity index		Weight Class	A	B	Validity index	
Under 58 kg	18	19.5	19	0.609	Under 49 kg	16	16.0	15	0.468
			20	0.615				16	0.436
Under 63 kg	20				Under 53 kg	17			
			21	0.622				17	0.412
Under 63 kg	20	20.1	19	0.479	Under 53 kg	17	17.5	17	0.605
			20	0.480				18	0.608
Under 68 kg	21				Under 57 kg	18			
			21	0.492				19	0.610
Under 68 kg	21	20.5	20	0.522	Under 57 kg	18	18.2	17	0.448
			21	0.516				18	0.434
Under 80 kg	23				Under 67 kg	20			
			22	0.526				19	0.426
Under 80 kg	23	20.0	19	0.459	Under 67 kg	20	17.3	16	0.530
			20	0.473				17	0.541
Over 87 kg	25				Over 67 kg	22			
			21	0.502				18	0.572

A: Kick impact standards officially established by the World Taekwondo Federation. B: Kick impact standards set in this study

## Discussion

The introduction of PSS to taekwondo was attractive to many coaches, athletes, and researchers. PSS is a measuring tool that determines whether a player scores during a match. Research on the validity and adequacy of the tool has been conducted continuously [10]. However, the criteria for determining the score in PSS, that is, research on the setting of the kick score impact is insufficient. Currently, the kick score impact setting is set by subjective judgment, thus leaving doubts. Therefore, this study was performed to confirm the adequacy of the setting of the impact of the kick protector for PSS.

First, as a result of confirming the form of impact of the PSS kick according to men's and women's weight class, it was confirmed that both men and women have similar patterns regardless of weight class. Especially, when comparing the average kick impact by weight class, it was determined that the difference in kick impact was not significant. In the current WT guidelines, the scoring impact for kicks is set higher for higher weight classes. Previous studies also reported that scoring frequency was relatively low in higher weight classes than in lower weight classes, pointing to the same problem of setting the scoring strength for kicks [3].

Furthermore, this study utilizes survey data to calculate and compare the PSS kick scoring impact reference point. As a result, it was confirmed that in both methods of calculating the reference point, the weighted kick score setting was set higher than the currently set kick score. The difference was 0.8 in the male lightweight division (under 58 kg) and 6 in the male heavyweight division (+ 80 kg). This indicates that the difference is greater in the heave weight division than in lightweight division. In fact, it is generally more logical to set a higher kick scoring impact for a higher weight class. However, considering that there is movement of the opponent player when being kicked and the kicks are defended by hand, setting a graded kick scoring impact is a matter of concern. In a taekwondo match, lower scoring may lead to lower interest, so further discussion will need to be made about the reference impact force used in scoring [8].

On the other hand, the kick scoring impact criteria suggested in the study results and the kick scoring impact set by the WT differs by 4 levels to 1 level. In fact, although a 1-level difference might seem small, it can still affect the score, and accordingly change the result. Since taekwondo has such characteristics, it is inevitable to emphasize the importance of setting the kick scoring impact of PSS.

Finally, the kick scoring impact setting in men's and women's different weight classes is dependent on the method and, thus, the limitations were that there are no clear standard of setting kick scoring impact (e.g. the kick scoring impact standard was under 53 kg: 15 levels, under 57 kg: 17 levels, and under 67 kg: 16 levels, showing that higher weight classes have a lower standard of kick scoring impact). In addition, only the Asian Games data were used, which can be interpreted as results for specific regions. Nevertheless, it is certain that this study provides basic data that could solve the problem of setting the kick scoring impact suggested by the WT. Also, this study attempted to identify the problem and solve it by using non-subjective data in setting the kick scoring impact, which may be used as important information in the future. Subsequent studies should focus on experts setting kick scoring impact according to the data results, which could be used as a more valid measure of athletic performance.

## Conclusion

This study confirmed the adequacy of setting the scoring impact of taekwondo kicks. The conclusions of this study are as follows. First, there was no difference in the average impact of kicks of taekwondo players by weight class. Second, setting up the kick scoring impact standards of taekwondo PSS by classifying the winning and non-winning groups, the kick scoring impact set by the WT was found to be high in all weight classes except under 58 kg. Lastly, comparing the settings of impact to score according to weight classes, the kick scoring impact standard set by the WT was higher in heavyweight (men's: under 80 kg, over 80 kg, women's: under 67 kg, over 67 kg) than in the lightweight (men's: under 58 kg, under 63 kg, under 68 kg, women's: under 49 kg, under 53 kg, under 57 kg).

## Data Availability

The datasets generated and/or analysed during the current study are not publicly available due to limitations of ethical approval involving the player data and anonymit but are available from the corresponding author on reasonable request.
